# Effectiveness of melatonin supplementation for improving sleep quality and disease severity in children with atopic dermatitis: a systematic review and meta-analysis

**DOI:** 10.3389/fmed.2025.1718859

**Published:** 2026-01-21

**Authors:** Fadi Alghamdi, Dhaii Y. Alzahrani, Renad F. Alharthy, Amal H. Abualola, Esraa A. Shaheen, Sadeen T. Shahbar, Renad O. Kalantan, Rose A. Alraddadi, Rami H. Jan, Waseem Alhawsawi, Bashaer H. Almahdi, Roaa E. Morya, Samar S. Alwafi, Nabeel S. Alamri, Ahmed K. Bakhsh

**Affiliations:** 1 King Fahad Armed Forces Hospital, Jeddah, Saudi Arabia; 2 College of Medicine, King Saud bin Abdulaziz University for Health Sciences, Jeddah, Saudi Arabia; 3 Department of Dermatology, King Fahad Medical City, Riyadh, Saudi Arabia; 4 Department of Dermatology, King Salman Medical City, Medina, Saudi Arabia; 5 Department of Dermatology, Hera General Hospital, Mecca, Saudi Arabia; 6 Department of Family Medicine, Ministry of National Guard-Health Affairs, King Abdulaziz Medical City, Jeddah, Saudi Arabia; 7 Department of Anesthesiology, Ministry of National Guard-Health Affairs, King Abdulaziz Medical City, Jeddah, Saudi Arabia; 8 Department of Dermatology, Ministry of National Guard-Health Affairs, King Abdulaziz Medical City, Jeddah, Saudi Arabia

**Keywords:** atopic dermatitis, children, disease severity, IgE, inflammation, melatonin, sleep quality

## Abstract

**Introduction:**

Atopic dermatitis (AD) is a chronic inflammatory skin condition causing pruritus, leading to sleep disturbance and poor quality of life, particularly in children. Effective adjunctive treatments addressing these issues are crucially needed.

**Objective:**

To evaluate the effects of melatonin supplementation on sleep quality and disease severity in children with atopic dermatitis through a systematic review and meta-analysis.

**Materials and methods:**

A systematic review with meta-analysis was conducted using three randomized controlled trials (RCTs). Eligible studies compared melatonin supplementation (3–6 mg/day) to placebo. Data was analyzed using a random-effects model with mean differences and standardized mean differences reported at 95% confidence intervals (CIs). Risk of bias was assessed using the Cochrane Risk of Bias Tool 2.0, and heterogeneity was evaluated using I^2^ statistics.

**Results:**

Melatonin significantly reduced sleep onset latency with a pooled standard mean difference of −0.63 (95% CI: −1.00 to −0.26, *p* = 0.0009) and improved disease severity with a pooled mean difference in SCORAD scores of −6.60 (95% CI: −10.11 to −3.10, *p* = 0.0002), but this did not exceed the MCID and total SCORAD was non-significant. Heterogeneity for these outcomes was minimal (I^2^ = 0–4%). However, melatonin had no significant effect on IgE levels (SMD = −0.19, 95% CI: −0.46 to 0.09, *p* = 0.18) or total sleep duration (mean difference = 18.29 min, 95% CI: −10.31 to 46.88, *p* = 0.21). No adverse events were reported, confirming its safety profile.

**Conclusion:**

Melatonin effectively improves sleep initiation in children with atopic dermatitis and may have modest effects on clinician-rated signs; the clinical importance remains uncertain. It appears safe, but further long-term studies are needed.

**Systematic review registration:**

https://www.crd.york.ac.uk/PROSPERO/view/CRD42023439385.

## Introduction

Atopic dermatitis (AD) also known as atopic eczema, is a chronic inflammatory skin condition predominantly affecting children. It presents with symptoms including intense pruritus, xerosis and recurrent eczematous rash, significantly impacting patients’ quality of life (1). Atopic dermatitis (AD) is a chronic inflammatory skin disease frequently accompanied by systemic manifestations such as pruritus-related sleep disturbance, creating a vicious cycle of itch, scratching, and disease worsening (2). Globally, an estimated 230 million people were affected in 2019, with most cases beginning in early childhood, and AD imposes a substantial burden on healthcare services, productivity, and quality of life for patients and caregivers (3–5).

Many studies have explored a wide range of therapeutic approaches to manage AD, from topical immunomodulators and phototherapy to conventional systemic immunosuppressants and more recent targeted systemic agents for moderate-to-severe pediatric disease (6, 7). Despite this expanding armamentarium, a substantial proportion of children continue to experience residual disease activity, treatment burden, and concerns about long-term safety, tolerability, or access (8). Emerging evidence has highlighted a potential role for melatonin, a hormone regulating circadian rhythms, in managing AD. Melatonin’s antioxidant and anti-inflammatory properties make it relevant not only to the disease’s cutaneous manifestations but also to the associated sleep disturbances (9).

Despite the existence of multiple positive data published in the literature demonstrating the efficacy of melatonin in AD, limited studies have systematically evaluated the effectiveness of melatonin supplementation in improving outcomes for children with AD. The existing studies have primarily focused on its role in sleep regulation (10), with sparse data on its impact on disease severity. Furthermore, variations in study designs, populations, and outcomes have hindered the synthesis of a clear conclusion on its efficacy.

To date, no comprehensive systematic review and meta-analysis has assessed the dual impact of melatonin on both sleep quality and disease severity in children with AD. This gap in the literature necessitates an in-depth investigation to guide clinical decision-making and provide evidence- based recommendations for this novel therapeutic approach. Our study aims to address this gap by systematically reviewing and analyzing randomized controlled trials (RCTs) evaluating the effects of melatonin supplementation in children with AD.

## Materials and methods

This study was conducted as a systematic review and meta-analysis (SRMA) to evaluate the effectiveness of melatonin supplementation in improving sleep quality and disease severity in children with AD. This systematic review and meta-analysis was prospectively registered in PROSPERO (Registration ID: CRD42023439385).

### Eligibility criteria

The eligibility criteria were established based on the Population, Intervention, Comparison, Outcomes and Study design (PICOS) framework. The population included children aged 1 to 18 years with physician-diagnosed AD involving at least 5% of the total body surface area. The intervention of interest was melatonin supplementation, with placebo as the comparison. The primary outcomes included AD severity measured using the SCORAD score, sleep quality, quality of life, melatonin levels (urinary or serum), serum IgE levels and adverse events. Only RCTs published in English were included. Studies involving participants with systemic illnesses, documented sleep disorders or neuropsychiatric conditions, as well as those receiving medications like corticosteroids, immunosuppressants or antihistamines, were excluded. Non-RCTs and studies with unavailable full-texts were also excluded.

### Literature search strategy

A systematic search was conducted using multiple databases, including Medline, Embase, the Cochrane Central Register of Controlled Trials (CENTRAL), Scopus and Clinicaltrials.gov, to identify relevant studies until September 2025. The search strategy combined keywords and MeSH terms related to atopic dermatitis (e.g., “Dermatitis, Atopic,” “eczema”) and melatonin (e.g., “melatonin supplementation”) with filters for RCTs and English language, while excluding conference abstracts and unpublished trials. The final search was performed, and duplicate records were removed before screening. The full Boolean search strategies for each database are provided in Supplementary Table 1.

### Flow of studies

**Figure 1:**
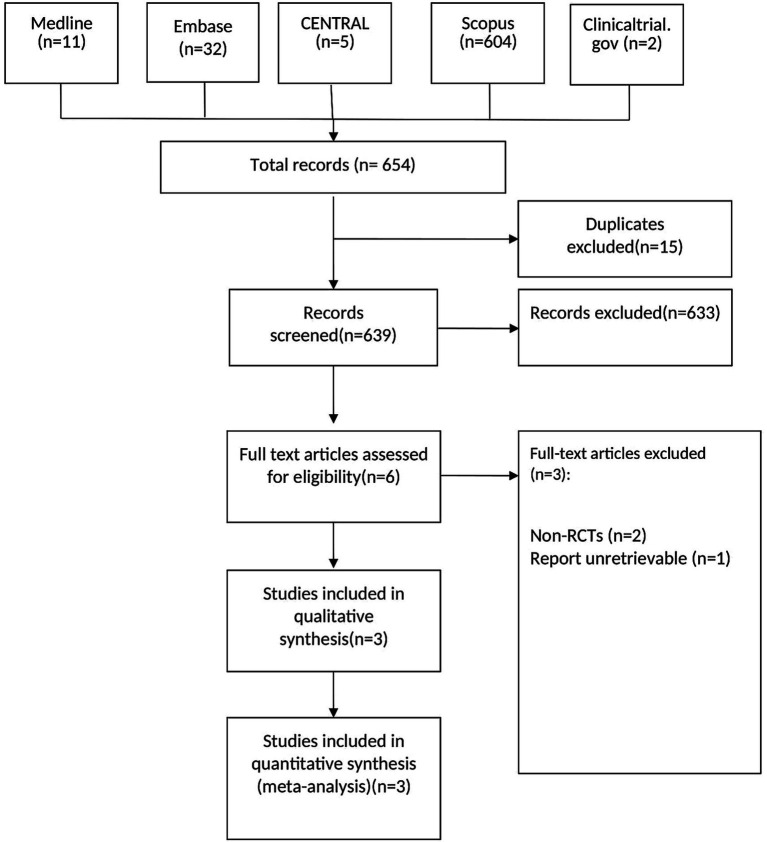
PRISMA chart showing selection and inclusion of studies (27).

The initial search yielded 654 records, of which 15 duplicates were excluded. After screening 639 records, 633 were excluded based on eligibility criteria. Full-text assessments were performed for six articles, three of which were excluded (two non-RCTs and one due to an unretrievable report). Finally, three RCTs were included in the qualitative and quantitative synthesis (Figure 1).

### Study selection and data extraction

Two independent reviewers screened titles and abstracts for eligibility with acceptable inter-reliability (Cohen’s Kappa = 0.78). Full-text assessments were conducted for potentially eligible studies. Disagreements were resolved through discussion with a third reviewer. Data from included studies were extracted using a standardized Excel sheet, capturing details such as study characteristics, demographics, intervention dosages, follow-up durations and outcomes reported (SCORAD scores, sleep quality, QoL, serum/urinary melatonin levels, serum IgE levels and adverse events).

### Risk of bias assessment

The risk of bias in the included studies was assessed using the Cochrane Risk of Bias Tool 2.0 (11). This tool evaluates domains such as randomization, deviations from intended interventions, missing outcome data, measurement of outcomes and selection of reported results. A visual summary and percentage distribution of the risk of bias were generated.

**Figure 2:**
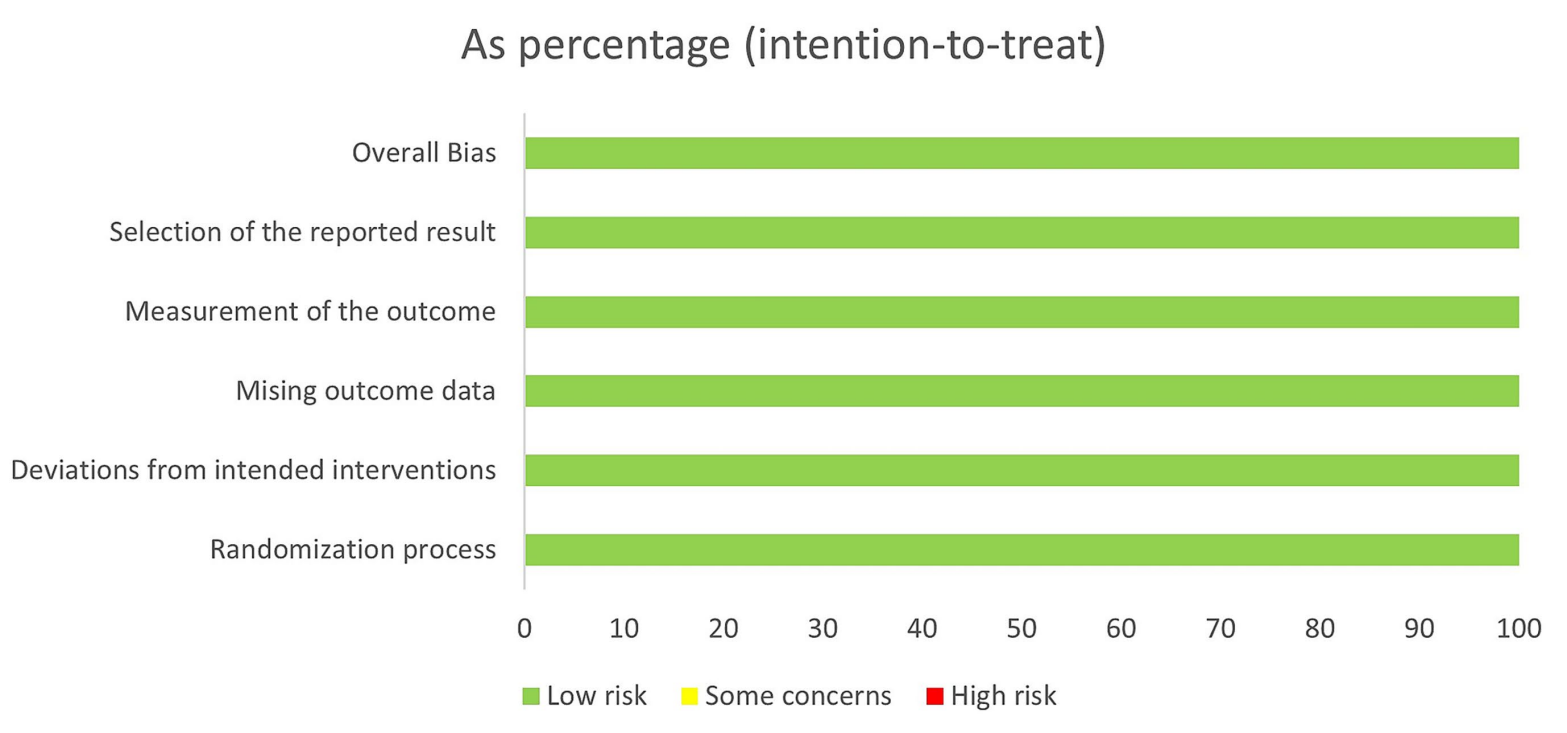
Risk of bias as percentage (intention-to-treat).

**Figure 3:**
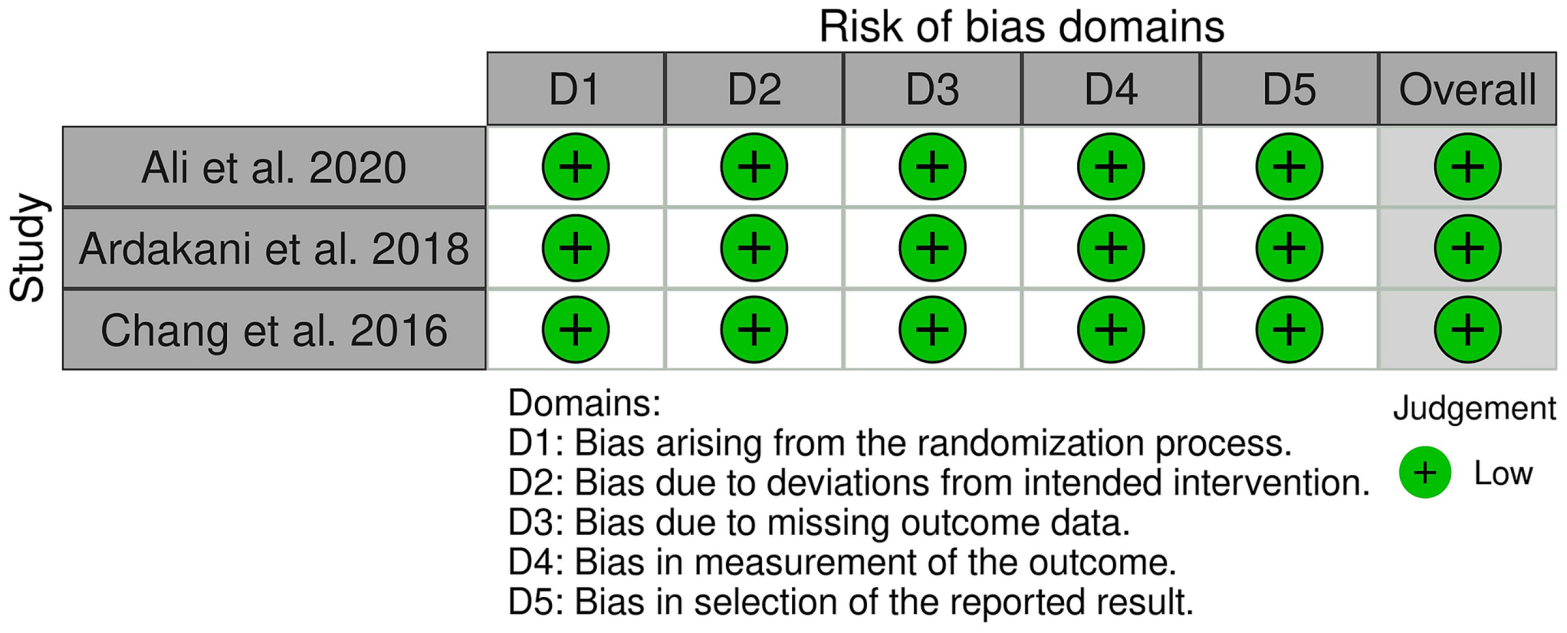
Risk of bias domains.

Figure 2 shows the distribution of risk levels across several domains of bias evaluations. All domains—Overall Bias, Selection of the Reported Result, Measurement of the Outcome, Missing Outcome Data, Deviations from Intended Interventions, and Randomization Process—are marked as having a low risk of bias, represented by the color green. This uniformity suggests that the studies incorporated in the meta-analysis are methodologically sound, with systematic application of treatment protocols and consistent reporting across all trials. The absence of ‘some concerns’ or ‘high risk’ levels in any domain indicates that the likelihood of bias affecting the study outcomes is minimal, bolstering confidence in the meta-analysis results. However, the evidence base remains constrained by the small number of RCTs and modest sample sizes, and one included trial has an Expression of Concern; therefore, conclusions should be interpreted cautiously and are not sufficient to support firm clinical recommendations without confirmation in larger, well-conducted studies. Figure 3 displays a comprehensive assessment of risk of bias across five specific domains for three studies included in a meta-analysis. The studies evaluated, Ali et al. (12), Ardakani et al. (13), and Chang et al. (14), all exhibit uniformly low risk across the domains of bias from the randomization process (D1), deviations from intended interventions (D2), missing outcome data (D3), measurement of the outcome (D4), and selection of the reported result (D5). Each domain is marked with a green plus symbol, indicating a low risk of bias, which contributes to the overall judgment of low risk for each study. This uniform low-risk evaluation across multiple critical domains suggests that the studies are methodologically sound and reliable, supporting the validity of the meta-analysis conclusions drawn from these data.

### Statistical analysis

The meta-analysis was performed using RevMan version 5.3, employing a random-effects model to account for variability across studies. Results were reported as risk ratios (RR) for categorical outcomes with 95% confidence intervals (CIs). Heterogeneity was assessed using the I^2^ statistic and the Chi^2^ test, with a significance threshold of *p* < 0.05. When all trials used the same measurement scale, treatment effects were summarized as mean differences (MD); when different scales were used across studies, we used standardized mean differences (SMD). The same effect size metric was applied consistently for each outcome across text, tables, and figures. In view of the Expression of Concern issued for the Ardakani et al. trial (13), we also considered the impact of excluding this study on the robustness of our findings. However, for key outcomes such as objective SCORAD, removing Ardakani et al. would leave only a single small trial, precluding a meaningful re-estimation of pooled effects and reinforcing the need for cautious interpretation of disease-severity outcomes. Planned subgroup analyses by melatonin dose and treatment duration were not feasible due to the limited number of trials and inconsistent reporting.

### Quality of evidence

The overall certainty of evidence for each outcome was graded using the GRADE (Grading of Recommendations, Assessment, Development, and Evaluation) framework. This approach considers factors such as study limitations, inconsistency, indirectness, imprecision and publication bias (Supplementary Table 2).

## Results

### Characteristics of the included studies

Three studies included in this analysis examined the effects of melatonin supplementation in children with atopic dermatitis, involving a total of 234 participants evenly distributed between intervention and placebo groups. The mean ages of children in the melatonin and placebo groups, ranges from approximately 5 to 9 years, with standard deviations (SD) indicating moderate variability within the age groups. Gender representation was similar between the two groups, with slightly more females than males but no significant imbalance overall (Table 1).

**Table 1:** Study characteristics and participants demographics.

First author’s last name, year of publication	Study arms	Number of participants	Age, mean (SD)	Gender (female), n	Gender (Male), n
Ali et al., 2020 (12)	Melatonin	43	5.46 (4.7)	19	24
	3 mg/d				
	Placebo	43	5.23 (2.1)	25	18
Ardakani et al., 2018 (13)	Melatonin 6 mg/d	35	8.9 (2.1)	19	16
	Placebo	35	8.4 (2.2)	17	18
Chang et al., 2016 (14)	Melatonin	24	7.6 (4)	13	11
	3 mg/d				
	Placebo	24	7.3 (3.5)	10	14

### Change in atopic dermatitis severity (SCORAD)

**Figure 4:**
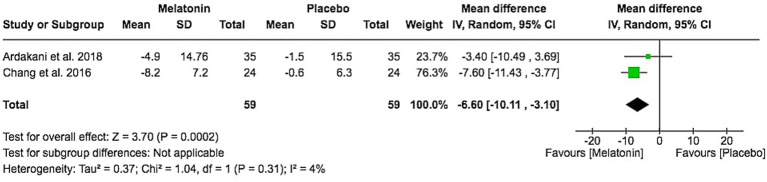
Objective SCORAD score change from baseline.

Two trials [Ardakani et al. 2018 (13) and Chang et al. 2016 (14)] reported change in objective SCORAD from baseline. The pooled mean difference showed a significant reduction in objective SCORAD with melatonin compared to placebo (MD − 6.60, 95% CI − 10.11 to −3.10; Z = 3.70, *p* = 0.0002), with minimal heterogeneity (I^2^ = 4%; Figure 4). Objective SCORAD mainly reflects clinician-assessed signs such as erythema, edema, and lichenification. Although the mean change did not exceed the minimal clinically important difference (MCID) of 8.2 points for objective SCORAD (15), this indicates a statistically detectable improvement in visible disease activity, while the magnitude of benefit is modest and its clinical relevance remains uncertain.

**Figure 5:**
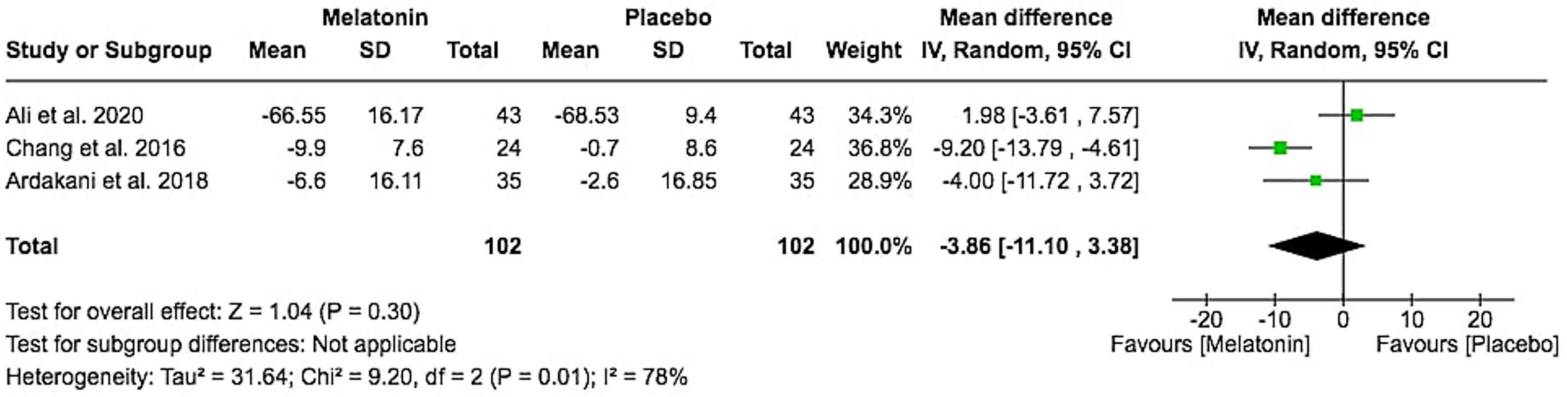
SCORAD score change from baseline.

A separate meta-analysis including three studies evaluated change in total SCORAD, which combines objective signs with patient-reported symptoms such as pruritus and sleep loss. The pooled mean difference favored melatonin but did not reach statistical significance (MD − 3.86, 95% CI − 11.10 to 3.38; Z = 1.04, *p* = 0.30), with high heterogeneity (I^2^ = 78%; Figure 5), and did not reach the MCID of 8.7 points for total SCORAD (15). This suggests that, at the doses and durations studied, any improvements in clinician-assessed signs may not consistently translate into clinically important changes in the combined signs-and-symptoms burden as perceived by patients and caregivers.

### Change in IgE levels

**Figure 6:**
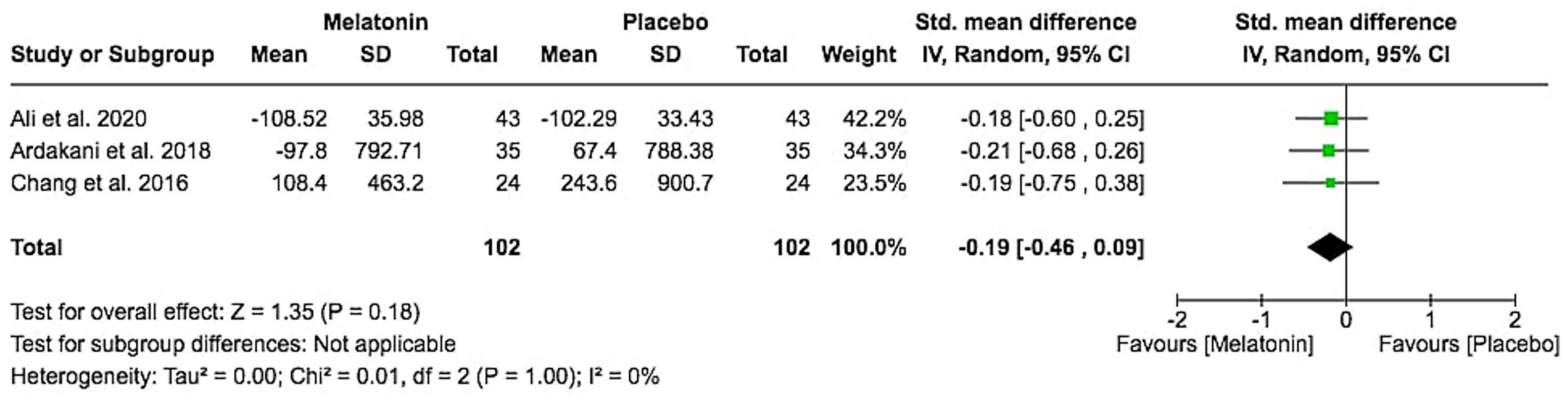
IgE level change from baseline.

Three trials reported changes in total IgE from baseline. The pooled standardized mean difference showed a small, non-significant reduction in IgE with melatonin compared to placebo (SMD − 0.19, 95% CI − 0.46 to 0.09; Z = 1.35, *p* = 0.18), with low heterogeneity (Figure 6). Overall, melatonin did not have a clear effect on IgE levels.

### Change in sleep onset latency

**Figure 7:**
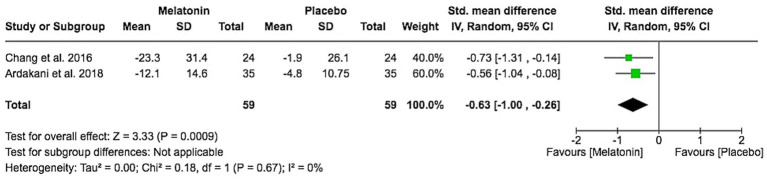
Sleep onset latency change from baseline.

Two studies reported change in sleep onset latency. Melatonin significantly reduced sleep onset latency compared with placebo (SMD − 0.63, 95% CI − 1.00 to −0.26; Z = 3.33, *p* = 0.0009), with no observed heterogeneity (I^2^ = 0%; Figure 7). This suggests a consistent beneficial effect of melatonin on the time taken to fall asleep.

### Change in total sleep time

**Figure 8:**
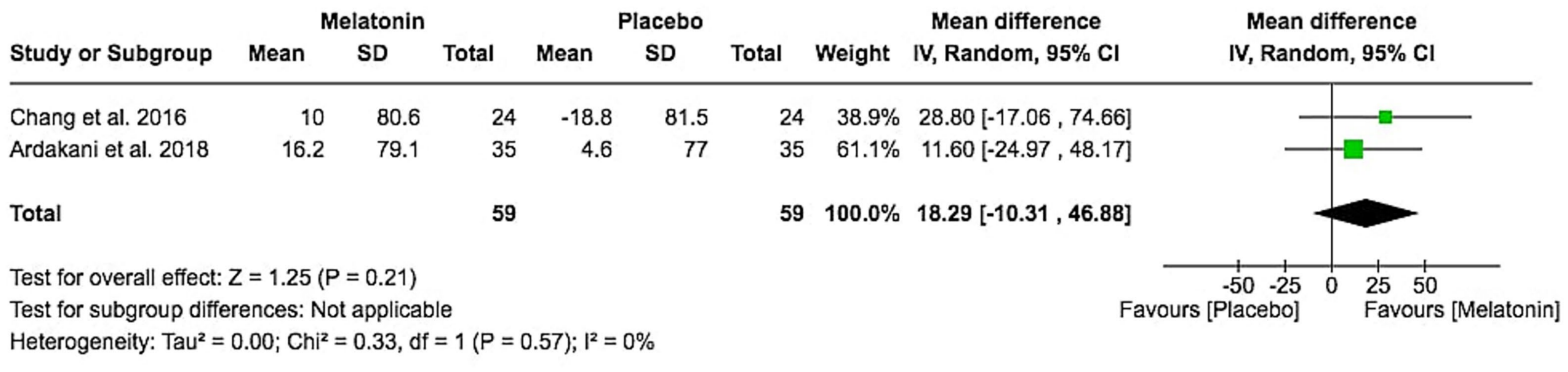
Total sleep time change from baseline.

Two studies assessed total sleep time. The pooled mean difference indicated a non-significant increase in total sleep time with melatonin (MD 18.29 min, 95% CI − 10.31 to 46.88; Z = 1.25, *p* = 0.21), with no heterogeneity (I^2^ = 0%; Figure 8). Thus, melatonin may modestly increase total sleep duration, but the effect was not statistically robust.

## Discussion

Atopic dermatitis in children is frequently complicated by sleep disturbance, which contributes to impaired quality of life and worse symptom control. In this context, our systematic review and meta-analysis evaluated whether melatonin supplementation improves both sleep-related outcomes and disease severity compared with placebo in pediatric AD.

Across two RCTs including 117 participants, melatonin significantly shortened sleep onset latency with a moderate standardized effect (SMD − 0.63, 95% CI − 1.00 to −0.26) and no heterogeneity (I^2^ = 0%), indicating a consistent benefit on the time it takes children to fall asleep. This is consistent with melatonin’s established role in circadian regulation through its action on receptors in the suprachiasmatic nucleus (16) and aligns with previous work showing improvements in sleep onset and nighttime awakenings in children with AD or sleep disorders (17–20). In contrast, the pooled increase in total sleep time (MD 18.29 min, 95% CI − 10.31 to 46.88) was modest and non-significant, suggesting that melatonin primarily facilitates sleep initiation rather than prolonging overall sleep duration (18–20).

The pharmacokinetic profile and chronobiologic actions of melatonin help explain this pattern of results. Orally administered melatonin is absorbed relatively rapidly, with peak concentrations occurring within the first hours after ingestion and a short elimination half-life, providing a strong but time-limited signal to initiate sleep (16–18). In addition to its acute soporific effects, melatonin interacts with MT1 and MT2 receptors in the suprachiasmatic nucleus, where it can phase-shift circadian rhythms and consolidate the sleep–wake cycle (13, 16–20). In the short trials included here, the predominant clinical effect appears to be on sleep onset latency, consistent with this acute pharmacodynamic window, whereas any longer-term circadian stabilization or downstream benefits on total sleep time or disease control may require longer treatment duration, different timing, or modified-release formulations, which were not evaluated in the available RCTs (13, 18–20).

For disease severity, melatonin produced a statistically significant reduction in objective SCORAD (MD − 6.60, 95% CI − 10.11 to −3.10; I^2^ = 4%) across two trials. Objective SCORAD primarily captures clinician-assessed signs (erythema, excoriation, lichenification), so this finding suggests a measurable improvement in visible skin manifestations. However, the magnitude of change remains below the published MCID threshold of 8.2 points for objective SCORAD (15), meaning that the statistical signal may not correspond to a clearly noticeable clinical improvement at the individual patient level. In contrast, total SCORAD, which integrates both objective signs and subjective symptoms such as pruritus and sleep loss, showed a non-significant pooled MD (−3.86, 95% CI − 11.10 to 3.38) with substantial heterogeneity (I^2^ = 78%) and did not reach the MCID of 8.7 points (15). The discordance between objective and total SCORAD suggests that modest improvements in clinician-rated signs do not consistently translate into clinically important changes in the combined signs-and-symptoms burden perceived by patients and caregivers. At the doses and treatment durations studied, melatonin therefore appears to have, at best, a modest and clinically uncertain effect on overall AD severity, even though it provides a more robust benefit for sleep initiation.

Objective SCORAD reflects clinician-rated signs (e.g., erythema, edema/papulation, excoriation, lichenification, dryness), whereas total SCORAD additionally incorporates subjective symptoms, particularly pruritus and sleep loss, that are more sensitive to contextual factors and may not change in parallel with visible signs (15). This construct difference likely contributes to the more consistent signal for objective SCORAD (low heterogeneity) compared with total SCORAD, where heterogeneity was substantial (I^2^ = 78%) and the pooled effect was not statistically significant.

Several trial-level factors may explain this heterogeneity in total SCORAD: (i) differences in baseline disease severity and baseline itch/sleep impairment, which can influence the magnitude of change and regression-to-the-mean; (ii) variability in melatonin dose (3 mg/day vs. 6 mg/day) and timing of administration, which may preferentially affect sleep initiation rather than daytime itch; (iii) differences in background care and allowed concomitant therapies (e.g., emollient use and rescue treatments), which may differentially improve subjective symptoms; and (iv) non-uniform outcome assessment time points and follow-up duration, which can change the observed effect if symptom improvement occurs earlier or later than skin-sign improvement.

An important caveat is that the largest improvement in SCORAD was reported in Ardakani et al. 2018, for which an expression of concern has been issued (13). This trial contributes substantially to the disease-severity signal and may overestimate the true benefit of melatonin. A sensitivity analysis excluding Ardakani et al. would leave only a single small RCT for objective SCORAD, precluding a robust re-estimation of pooled effects and further lowering confidence in the severity signal. These considerations are reflected in our GRADE assessment, which rated the certainty of evidence for disease severity as low, despite biologically plausible anti-inflammatory and antioxidant actions of melatonin on the skin (13, 21–23).

Melatonin did not show a clear effect on systemic allergy markers. The pooled SMD for total IgE change (−0.19, 95% CI − 0.46 to 0.09) indicated a small, non-significant reduction with low heterogeneity. This is compatible with the possibility that, within the short treatment windows examined, melatonin’s benefits are mediated mainly by local or neuro-immune pathways affecting pruritus and sleep, rather than by large changes in systemic IgE (10, 24). Whether longer treatment duration, higher dosing, or different formulations (13) can influence systemic biomarkers remains uncertain and warrants further study.

Safety data were reassuring but limited. Across three RCTs (234 participants), no serious adverse events attributable to melatonin were reported, and non-serious adverse events were infrequent and similar between melatonin and placebo, in line with previous pediatric studies supporting a favorable short-term safety profile even at relatively higher doses (13, 14, 25). However, adverse events were collected and reported inconsistently, follow-up was short, and sample sizes were modest, so rare or long-term harms cannot be excluded.

Taken together, our findings support melatonin as a potentially useful adjunctive option for children with AD who experience prominent difficulties with sleep initiation, offering a statistically robust and clinically plausible benefit on sleep onset. In contrast, its impact on disease severity and systemic markers appears modest, of uncertain clinical importance, and sensitive to concerns around one key trial. In line with the broader shift toward non-pharmacologic and adjunctive strategies in AD management (10, 26), melatonin may be considered as an add-on in selected patients, but routine use for disease control cannot be recommended on current evidence. Larger and rigorously conducted RCTs with standardized severity and sleep outcomes, detailed safety reporting, and predefined subgroup analyses are needed to clarify the magnitude and durability of both dermatologic and sleep benefits and to identify which children are most likely to benefit, or to evaluate melatonin as an adjunct to standard-of-care regimens (including topical corticosteroids and other commonly used therapies) to better define real-world effectiveness and safety. Future trials should also standardize dosing schedules and follow-up windows and report outcomes consistently to enable dose- and duration-specific meta-analytic estimates. Given the chronic, relapsing nature of AD, longer-term trials are needed to determine whether sleep and skin benefits persist and to monitor potential cumulative or developmental effects of prolonged melatonin exposure.

### Strengths and limitations

One of the key strengths of this analysis is the inclusion of studies with low risk of bias, as assessed using the Cochrane Risk of Bias Tool. The uniform low-risk evaluation across domains enhances the reliability of the findings. However, the modest sample sizes, and the relatively short follow-up periods represent important limitations, which precluded a reliable assessment of publication bias or the evaluation of long-term control or relapse. Importantly, only three RCTs were eligible (total *N* = 234), and individual trial sample sizes were modest (*N* = 48–86), limiting statistical power, widening uncertainty around effect estimates, and reducing generalizability across the full spectrum of pediatric ages and AD severities. These constraints also limited our ability to explore age- or severity-based subgroup effects and may have contributed to imprecision for several outcomes. An additional and potentially serious limitation is that one of the three included RCTs, Ardakani et al. 2018, is the subject of an Expression of Concern (13). Because this trial contributes substantially to the evidence on disease-severity outcomes, any unresolved issues affecting its validity could bias the pooled estimates in favour of melatonin. We considered the impact of excluding Ardakani et al. in a sensitivity analysis; however, for key outcomes this would leave only a single small RCT, making any re-estimated effect sizes unstable and further reducing the certainty of the evidence.

There was also clinical heterogeneity in melatonin regimens, including differences in dose (3 mg/day vs. 6 mg/day) and variability in treatment duration and follow-up schedules across trials. Because only three RCTs were available and outcome reporting was not uniform, we could not perform prespecified subgroup analyses by dose or duration, nor assess dose–response relationships. Therefore, the optimal dosing strategy, timing, and duration of melatonin supplementation for pediatric AD remain uncertain. The substantial heterogeneity observed for total SCORAD limits interpretability of the pooled estimate and reduces confidence in a consistent effect on patient-perceived symptom burden. Also, several trials excluded children using common AD medications (corticosteroids, immunosuppressants, antihistamines), which improves internal validity but limits real-world applicability; therefore, results may not generalize to typical moderate–severe patients treated with standard concomitant therapies. Restricting inclusion to published, English-language RCTs also raises the possibility of language and publication bias. Long-term studies with larger sample sizes and standardized outcome measures are needed to assess the sustained benefits and potential long-term safety of melatonin supplementation in this population. Finally, adverse events and tolerability were inadequately and inconsistently reported across studies, limiting our ability to synthesize safety outcomes or draw firm conclusions about tolerability.

## Conclusion

Melatonin improves sleep initiation and may have modest effects on clinician-rated signs; the clinical importance remains uncertain. This conclusion is based on three small RCTs (total *N* = 234), in which observed SCORAD reductions did not exceed established MCID thresholds and total SCORAD was non-significant. Larger, longer-term trials with broader age and severity representation are needed to confirm durability of benefit and long-term safety.

## Data Availability

The original contributions presented in the study are included in the article/Supplementary material, further inquiries can be directed to the corresponding author.
