# Supplementary material for: Effectiveness of melatonin supplementation for improving sleep quality and disease severity in children with atopic dermatitis: a systematic review and meta-analysis
Source: Front Med (Lausanne). 2026 Jan 21;12:1718859. doi: 10.3389/fmed.2025.1718859 (PMC12867768; doi:10.3389/fmed.2025.1718859)
Supplement: Supplementary file 1 [file Data_Sheet_1.PDF]

Supplementary Table 1. Full Boolean Search Strategies for All Databases

Database	Search Date	Full Boolean Search Strategy	Results
Medline (via PubMed)	12 September 2025	atopic dermatitis (e.g., "Dermatitis, Atopic," "eczema") and melatonin (e.g., "melatonin supplementation") Filters: Humans, English.	11
Embase (via Elsevier)	13 September 2025	atopic dermatitis (e.g., "Dermatitis, Atopic," "eczema") and melatonin (e.g., "melatonin supplementation") AND [english]/lim AND [humans]/lim	32
Cochrane CENTRAL	13 September 2025	atopic dermatitis (e.g., "Dermatitis, Atopic," "eczema") and melatonin (e.g., "melatonin supplementation")	5
Scopus	14 September 2025	atopic dermatitis (e.g., "Dermatitis, Atopic," "eczema") and melatonin (e.g., "melatonin supplementation") AND (LIMIT-TO(LANGUAGE, "English"))	604
ClinicalTrials.gov	14 September 2025	Condition or disease: (“atopic dermatitis” OR eczema). Intervention: melatonin. Other terms: randomized OR placebo. Filters: Completed, Child (0–17 years), Interventional studies.	2
