# Supplementary material for: Effectiveness of melatonin supplementation for improving sleep quality and disease severity in children with atopic dermatitis: a systematic review and meta-analysis
Source: Front Med (Lausanne). 2026 Jan 21;12:1718859. doi: 10.3389/fmed.2025.1718859 (PMC12867768; doi:10.3389/fmed.2025.1718859)
Supplement: Supplementary file 2 [file Data_Sheet_2.PDF]

Supplementary Table 2. GRADE evidence profile

Outcome	Participants (studies, follow-up)	Risk of bias	Inconsistency	Indirectness	Imprecision	Publication bias	Overall certainty of evidence	Relative effect (95% CI)	Risk with placebo	Risk difference with melatonin
**Objective SCORAD – change from baseline (lower = better)**	118 participants (2 RCTs), 4–8 weeks	Not serious	Not serious	Not serious	**Serious[1]**	Not serious[4]	⊕⊕⊕◯ **Moderate**	MD −6.60 points (95% CI −10.11 to −3.10) in favour of melatonin	Not applicable	On average, melatonin results in a 6.6-point greater reduction in objective SCORAD compared with placebo
**Total SCORAD – change from baseline (lower = better)**	204 participants (3 RCTs), 4–8 weeks	Not serious	**Serious[3]**	Not serious	**Serious[2]**	Not serious[4]	⊕⊕◯◯ **Low**	MD −3.86 points (95% CI −11.10 to 3.38); effect uncertain	Not applicable	Melatonin may reduce total SCORAD by ~3.9 points more than placebo, but the CI includes no important difference
**Total IgE level – change from baseline (lower = better)**	204 participants (3 RCTs), 4–8 weeks	Not serious	Not serious	Not serious	**Serious[2]**	Not serious[4]	⊕⊕⊕◯ **Moderate**	SMD −0.19 (95% CI −0.46 to 0.09) in favour of melatonin	Not applicable	Melatonin probably results in little to no reduction in IgE levels compared with placebo
**Sleep onset latency – change from baseline (lower = better)**	118 participants (2 RCTs), 4–8 weeks	Not serious	Not serious	Not serious	**Serious[1]**	Not serious[4]	⊕⊕⊕◯ **Moderate**	SMD −0.63 (95% CI −1.00 to −0.26) in favour of melatonin	Not applicable	Melatonin probably reduces sleep onset latency compared with placebo by a moderate amount
**Total sleep time – change from baseline (higher = better)**	118 participants (2 RCTs), 4–8 weeks	Not serious	Not serious	Not serious	**Serious[2]**	Not serious[4]	⊕⊕⊕◯ **Moderate**	MD 18.29 minutes (95% CI −10.31 to 46.88); effect uncertain	Not applicable	Melatonin may increase total sleep time, but the CI includes no important difference

1. **Imprecision (small sample, CI excludes no effect):** downgraded one level because only 118 participants from two small trials contributed data; optimal information size is not met even though the confidence interval excludes no effect (objective SCORAD, sleep onset latency).

2. **Imprecision (CI crosses no effect):** downgraded one level because the confidence interval crosses the line of no effect and includes both a potentially important benefit and no important difference, with limited total sample size (total SCORAD, IgE, total sleep time).

3. **Inconsistency:** downgraded one level because heterogeneity for total SCORAD was substantial ($I^2 = 78\%$) with notable variation in effect sizes across trials.

4. **Publication bias:** not downgraded; the small number of RCTs precluded formal assessment (e.g., funnel plots), but there was no clear evidence of selective publication.
